# MicroRNA-125a is over-expressed in insulin target tissues in a spontaneous rat model of Type 2 Diabetes

**DOI:** 10.1186/1755-8794-2-54

**Published:** 2009-08-18

**Authors:** Blanca M Herrera, Helen E Lockstone, Jennifer M Taylor, Quin F Wills, Pamela J Kaisaki, Amy Barrett, Carme Camps, Christina Fernandez, Jiannis Ragoussis, Dominique Gauguier, Mark I McCarthy, Cecilia M Lindgren

**Affiliations:** 1 Wellcome Trust Centre for Human Genetics, University of Oxford, Roosevelt Drive, Oxford, OX3 7BN, UK; 2 Oxford Centre for Diabetes, Endocrinology and Metabolism, The Churchill Hospital, Headington, Oxford, OX3 7LJ, UK; 3 Department of Statistics, 1 South Parks Road, Oxford, OX1 3TG, UK; 4 INSERM U872, Centre de Recherche des Cordeliers, Rue de l'Ecole de Medecine, 75006 Paris, France

## Abstract

**Background:**

MicroRNAs (miRNAs) are non-coding RNA molecules involved in post-transcriptional control of gene expression of a wide number of genes, including those involved in glucose homeostasis. Type 2 diabetes (T2D) is characterized by hyperglycaemia and defects in insulin secretion and action at target tissues. We sought to establish differences in global miRNA expression in two insulin-target tissues from inbred rats of spontaneously diabetic and normoglycaemic strains.

**Methods:**

We used a miRNA microarray platform to measure global miRNA expression in two insulin-target tissues: liver and adipose tissue from inbred rats of spontaneously diabetic (Goto-Kakizaki [GK]) and normoglycaemic (Brown-Norway [BN]) strains which are extensively used in genetic studies of T2D. MiRNA data were integrated with gene expression data from the same rats to investigate how differentially expressed miRNAs affect the expression of predicted target gene transcripts.

**Results:**

The expression of 170 miRNAs was measured in liver and adipose tissue of GK and BN rats. Based on a *p*-value for differential expression between GK and BN, the most significant change in expression was observed for miR-125a in liver (FC = 5.61, *P *= 0.001, *P*_*adjusted *_= 0.10); this overexpression was validated using quantitative RT-PCR (FC = 13.15, *P *= 0.0005). MiR-125a also showed over-expression in the GK vs. BN analysis within adipose tissue (FC = 1.97, *P *= 0.078, *P*_*adjusted *_= 0.99), as did the previously reported miR-29a (FC = 1.51, *P *= 0.05, *P*_*adjusted *_= 0.99). *In-silico *tools assessing the biological role of predicted miR-125a target genes suggest an over-representation of genes involved in the MAPK signaling pathway. Gene expression analysis identified 1308 genes with significantly different expression between GK and BN rats (*P*_adjusted _< 0.05): 233 in liver and 1075 in adipose tissue. Pathways related to glucose and lipid metabolism were significantly over-represented among these genes. Enrichment analysis suggested that differentially expressed genes in GK compared to BN included more predicted miR-125a target genes than would be expected by chance in adipose tissue (FDR = 0.006 for up-regulated genes; FDR = 0.036 for down-regulated genes) but not in liver (FDR = 0.074 for up-regulated genes; FDR = 0.248 for down-regulated genes).

**Conclusion:**

MiR-125a is over-expressed in liver in hyperglycaemic GK rats relative to normoglycaemic BN rats, and our array data also suggest miR-125a is over-expressed in adipose tissue. We demonstrate the use of *in-silico *tools to provide the basis for further investigation of the potential role of miR-125a in T2D. In particular, the enrichment of predicted miR-125a target genes among differentially expressed genes has identified likely target genes and indicates that integrating global miRNA and mRNA expression data may give further insights into miRNA-mediated regulation of gene expression.

## Background

MicroRNAs (miRNAs) are short (~22 nucleotides) non-coding RNA molecules that regulate gene expression at a post-transcriptional level through sequence alignment mechanisms. MiRNA molecules bind to the 3' untranslated region (UTR) of their target mRNAs and can cause either mRNA degradation or translational repression, resulting in reduced protein expression [1] or translational activation depending on cell cycle stage [2]. Degradation of mRNA seems to be favoured if the binding occurs with perfect sequence complementarity and is widely observed in plant miRNAs [3,4]. A variety of studies have demonstrated that regulation at the mRNA level also occurs for animal miRNAs [5,6]. Microarray-based experiments have shown that overexpression of specific miRNAs in human cells down-regulates many transcripts predicted to bind the miRNA molecule [6-8]. Conversely, silencing of endogenous miR-122 in mice caused the preferential up-regulation of transcripts containing miR-122 binding sites [9].

MiRNA expression levels are thought to contribute to tissue-specific gene expression patterns [10] and computational approaches to integrating miRNA and gene expression data have provided insights into miRNA-mRNA interactions [11,12]. A single miRNA molecule can affect the expression of many target genes and therefore miRNAs are thought to be involved in the regulation of a wide variety of normal biological processes [13].

Type 2 diabetes (T2D) is characterized by hyperglycaemia that arises via combined defects in insulin secretion (beta-cell dysfunction) and insulin action (in target tissues like adipose tissue, liver and skeletal muscle). Specific miRNAs involved in various aspects of glucose and lipid metabolism have been identified in recent years [14,15]. In particular, using murine models, miR-9 and miR-375 are reported to be involved in regulation of insulin secretion [16,17], while miR-124a2 has recently been implicated in pancreatic beta-cell development and function [18]. Despite the growing evidence that miRNAs may be important in T2D, only one study of global miRNA expression in insulin target tissues has been published to date. He *et al*. (2007) [19] profiled miRNA expression in skeletal muscle in the Goto-Kakizaki (GK) rat, a well-characterized model of T2D, and compared it to normal Wistar rats; miR-29 paralogs were found to be over-expressed in the GK rat. The widely-studied GK rat has several features that make it a good model for T2D as hyperglycaemia appears as early as 2–4 weeks of age, and it is relatively lean [20]. In the present study, we aim to identify differential liver and adipose tissue miRNA expression in rats of the GK strain and a genetically distant normoglycaemic strain (Brown-Norway (BN)). The GK strain is extensively used to study genetic determinants of T2D phenotypes [21], while the BN strain is widely used for comparison with GK and most congenic strains with a BN background. Additionally, we assessed mRNA expression levels in the same tissues from the same rats to investigate the downstream effects of altered miRNA expression on target gene expression; the approach is illustrated in additional File 1 [Additional file 1].

## Methods

### Experimental animals

The GK rat is a lean and spontaneously type 2 diabetic animal, while the BN rat serves as a normoglycaemic control. Four-month old rats representative of the GK and BN colonies have been phenotyped previously. An intra-peritoneal glucose tolerance test (IPGTT) [22] was carried out after a 16–18 h fasting period, and plasma glucose concentrations were significantly higher in GK compared to BN rats [see Additional file 2]. The current experiments were performed using seven-month-old male rats: four from the GK strain and four from the BN strain, obtained from the Oxford colony (GK/Ox and BN/Ox), and maintained in accordance with national and institutional guidelines. Rats were fed standard laboratory chow pellets (B&K Universal, Hull, UK) and kept on 12 h light/dark cycles. Liver and adipose tissue samples were obtained from the rats, snap-frozen in liquid nitrogen and stored at -80°C. Total RNA was extracted from homogenized tissue samples using TRI-Reagent (Sigma, UK) according to the manufacturer's instructions for use in miRNA and mRNA experiments (see below). Animal procedures were approved by the ethical review panel of the University of Oxford and UK Home Office licences.

### Microarray analysis

#### Ambion miRNA arrays

MiRNAs were isolated and purified using mirVana miRNA isolation kit (Ambion). Poly-A tailing was added to 400 ng of each small RNA fraction using Ncode miRNA labelling system (Invitrogen), which also adds the fluorescent labels Alexa Fluor^® ^3 and Alexa Fluor^® ^5. The synthetic sequence control-1 (Ambion) was spiked in to each BN and GK sample in a ratio of 1:10, just before starting the labelling. For each tissue, eight independent samples (four GK and four BN) were combined in four two-colour hybridisations, with one GK and one BN sample hybridized to each array. Within each tissue, half the arrays had GK samples labelled with Alexa Fluor^® ^5 and the other half had BN samples labelled with Alexa Fluor^® ^5.

Samples were hybridised to Ambion mirVana™ miRNA Probe Set 1564v1 arrays for 4 h at 62°C in dark conditions. The array contains 384 unique probes with 16 replicates of each probe. Each probe is 42 to 46 nucleotides (nt) long, of which an 18 to 24 nt segment targets a specific miRNA. MiRNA sequences are highly conserved between species, and the Ambion array includes probes for human, rat and mouse miRNAs. A total of 170 probes on the array are able to detect rat miRNAs and these were included in subsequent analyses. After hybridization, arrays were washed and scanned using the GenePix 4000B scanner (Axon Instruments [Molecular Devices]). GenePix Pro 4.0 software (Axon Instruments [Molecular Devices]), was used to obtain raw probe intensities.

#### Statistical analysis of Ambion miRNA data

Raw data were imported into the R statistical package [23] and processed using the 'marray' package from Bioconductor [24]. Raw intensities were adjusted by subtracting the background intensity from each probe, and the data were then summarised by taking the median intensity across the 16 replicates of each probe. Data were normalized using the loess regression-based method within arrays to correct for any intensity-dependent dye bias [25]. A between-array normalisation was not required as all arrays showed comparable M-ratio distributions. Due to expected tissue differences in miRNA expression, differential expression between GK and BN rats was assessed in adipose tissue and liver samples separately. The experimental design (see above) generated M-ratios (log-ratio of red and green intensities) representing the log ratio of expression in the two strains. The M-ratios could therefore be used to investigate strain differences, the main objective of this study. We used the limma package from BioConductor [26] to fit a linear model (including a dye effect) to the M-ratio data from the 4 arrays corresponding to each tissue. This generated a list of miRNA probes ranked by p-value for the evidence for differential expression between the GK and BN rats. Raw p-values were adjusted using the method of Benjamini and Hochberg [27] to control the false discovery rate (FDR). Differential expression between liver and adipose tissue could also be investigated using the A-ratios, which represent the average intensity (log scale) of the GK and BN samples hybridised to each array. Assuming strain differences are small in comparison to tissue differences, the fold change (liver vs. adipose tissue) can be estimated as the difference in mean A-ratio between the 4 liver and 4 adipose tissue arrays.

#### Illumina mRNA arrays

Gene expression profiling of liver and adipose tissue samples from the same rats investigated in the miRNA experiment was performed using Sentrix^® ^BeadChip RatRef-12 v1 Whole-Genome Gene Expression Arrays (Illumina Inc., San Diego, California, USA), which contain 22,523 oligonucleotides probes (replicated an average of 30 times). Double-stranded cDNA and purified biotin-labelled cRNA were synthesised from 300 ng high quality total RNA using the Illumina^® ^TotalPrep RNA Amplification Kit (Ambion Inc., Austin, Texas, USA). cRNA concentrations were determined using a NanoDrop spectrophotometer, and cRNA quality and integrity were assessed using an Agilent 2100 Bioanalyser (Agilent Technologies, Waldbronn, Germany). Hybridisations onto Sentrix^® ^BeadChip RatRef-12 v1 arrays were carried out using 750 ng of each biotinylated cRNA in a 58°C hybridisation oven for 18 hours. Following washing and staining with Streptavidin-Cy3, the BeadChip Arrays were scanned on the Illumina^® ^BeadArray Reader (Illumina Inc., San Diego, USA). The quality of the resulting data was checked using the Illumina^® ^BeadStudio Application software

#### Statistical analysis of Illumina mRNA data

Raw intensity data were imported into the R statistical package [23] for further processing. First, an array-specific background measure, based on the average signal from negative control probes, was subtracted from all probes on the array. Data were then transformed and normalized using the vsn2 BioConductor package [28]. Hierarchical clustering showed samples clustered primarily according to tissue, with GK and BN samples forming two sub-clusters within the adipose tissue cluster. Differential expression between the GK and BN strains was assessed in liver and adipose tissue samples separately using the limma package from BioConductor [26]. Raw p-values were adjusted to control the false discovery rate (FDR) using the method of Benjamini and Hochberg [27]; adjusted p-values below 0.05 were considered significant.

The entire datasets (for miRNA and mRNA) described here are available from the Gene Expression Omnibus (GEO, ) through series accession number GSE17060.

#### Quantitative real-time PCR validation methods

Quantitative real-time PCR was carried out on the remainder of RNA available from liver tissues using ABI's TaqMan microRNA assays (Applied Biosystems (ABI), Warrington, Cheshire, UK). Briefly, reverse transcription (RT) was carried out in a total reaction volume of 15 μl containing 5 μl of total RNA (10 ng/ul), 3 μl of reverse transcription primer, 1.50 μl of 10× RT buffer, 1.00 μl MultiScribe Reverse Transcriptase (50 U/μl), 0.15 μl of 100 mM dNTPs (with dTTP), 0.19 μl of RNase Inhibitor, 20 U/μl and 4.16 μl of nuclease-free water. Reactions were incubated as per manufacturer's recommendation. PCRs were performed in triplicate, the 20-μL PCR reaction contained: 1.33 μL RT product, 10 μL 2× PCR Master Mix, 1-μL microRNA primer (ABI, UK) and 7.67 μl of nuclease-free water. The reactions were incubated at 95°C for 10 min, followed by 40 cycles of 95°C for 15 s, 60°C for 35 s. The highly conserved snoRNA and 4.5S RNA(H) (rat) were used as normalizing endogenous controls. Fold changes (FC) in expression were calculated using the 2^-(ΔΔCt) ^method [29]. Stocks of adipose tissue were depleted when used in the miRNA and mRNA studies, which did not allow for validation using RT-PCR.

### *In-silico *prediction of miRNA target genes

*In-silico *prediction of miRNA target genes was carried out using three different prediction algorithms: TargetScan v4.1 [30], PicTar [31] and miRanda [32]. The first two algorithms predict binding sites preferentially based on the 5' end of the 22 nt miRNA sequence, while miRanda predictions are more sensitive to the sequence at the 3' end. Each prediction algorithm generally identifies hundreds of potential target genes for any single miRNA and there is relatively little overlap between different algorithms. However, TargetScan and PicTar show a higher degree of overlap than either does with miRanda [Additional file 3], as might be expected based on the approaches implemented by the different algorithms. These predicted target lists may also contain a significant proportion of false-positives i.e. the sequences align, but no functional miRNA-mRNA interaction occurs *in vivo *[33]. With little experimental data, it is difficult to determine which algorithm performs best; thus, all analyses were performed with each list separately as well as with the subset of target genes predicted by at least two of the three algorithms. The latter list may be more likely to include a higher proportion of true target genes than any one list alone, but will be biased towards TargetScan/PicTar predictions.

### *In-silico *functional analysis

Biological pathways defined by KEGG [34] that are enriched among the predicted target genes of miR-125a and the lists of differentially expressed mRNAs found in liver and adipose tissue were identified using the GENECODIS software [35]. This profiling tool uses the hypergeometric distribution to determine whether individual pathways or combinations of pathways are significantly over-represented among the genes of interest. P-values computed for each pathway were adjusted using the method of Benjamini and Hochberg [36] to control the false discovery rate (FDR) and adjusted p-values < 0.05 were considered significant.

### Enrichment of miR-125a target genes among differentially expressed genes

Using the mRNA expression data, we investigated whether predicted miR-125a target genes were differentially expressed in liver or adipose tissue. Since miRNAs can cause degradation of target mRNAs, over-expression of miR-125a in both tissues in the GK rat may down-regulate its target genes in GK relative to BN. As described above, 3 different algorithms were used to generate target gene lists for miR-125a. Each of these lists, together with the subset of 152 genes predicted by more than one algorithm, was tested in this analysis. Only target genes present on the array were included, which typically reduced the list of targets by about 40% (Table 1). We first calculated how many genes in each of the four target gene lists were among the significantly down-regulated genes in adipose tissue (n = 598) and liver (n = 138). Then, a permutation test was used to assess whether the observed overlap was greater than that expected by chance. Specifically, we selected 10,000 random sets of genes that were not predicted targets of miR-125a, with each set containing the same number of genes as predicted targets. The overlap with the down-regulated genes in adipose tissue and liver was calculated for each of the 10,000 random gene sets. The proportion of random gene sets showing equal or greater overlap as the set of miR-125a target genes was used to estimate the false discovery rate. This analysis was carried out 8 times in total (for each of four lists in two tissues) and repeated exactly as above for the up-regulated genes.

**Table 1:** Overlap between predicted miR-125a target genes and genes significantly altered in GK compared to BN rats in adipose tissue and in liver.

	**miRanda**		**PicTar**		**TargetScan**		**>1 Algorithm**	
Predicted miR-125a target genes	975		350		165		152	
Number genes tested	536		231		94		114	

	**Overlap**	**FDR**	**Overlap**	**FDR**	**Overlap**	**FDR**	**Overlap**	**FDR**

Liver up-regulated (95 genes)	5	0.074	1	0.63	1	0.33	1	0.40
Liver down-regulated (138 genes)	5	0.248	2	0.43	0	1.00	0	1.00
Adipose up-regulated (477 genes)	**21**	**0.006**	5	0.57	1	0.87	3	0.46
Adipose down-regulated (598 genes)	**22**	**0.036**	7	0.44	1	0.92	0	1.00

The number of predicted miR-125a target genes found among the significantly up- and down-regulated genes in each tissue is shown for each algorithm and for the subset of targets predicted by more than one algorithm. In each case, the significance of the overlap (estimated false discovery rate) is calculated as the proportion of 10,000 random sets of non-miR-125 target genes of the same size showing equal or greater overlap.

## Results

### Differences in miRNA expression between GK and BN rats

**Figure 1:**
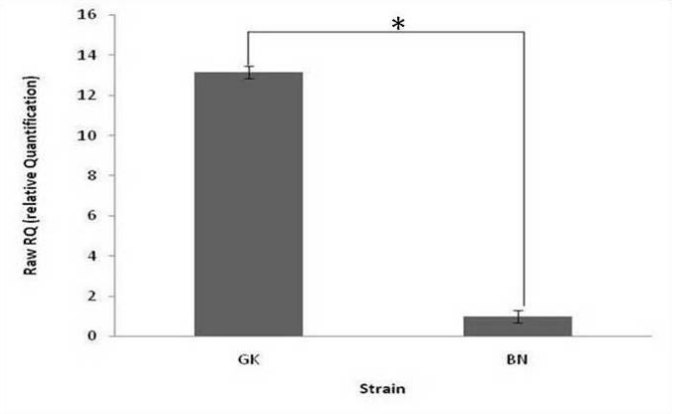
**Relative expression of miR-125a in liver from diabetic GK rats compared to BN rats**. * *P *= 0.0005 (miR-125a expression normalized against snoRNA and 4.5S).

The expression of 170 miRNAs in adipose tissue and liver from hyperglycaemic GK and normoglycaemic BN rats was detected using two-colour microarray technology (Ambion mirVana™ miRNA Probe Set 1564v1). As expected tissue-specific patterns of miRNA expression were detected [Additional file 4]; all subsequent GK vs. BN analyses were carried out within tissues. Differences in miRNA expression across strains were ranked by p-value from an analysis of differential expression using the limma package [26] from BioConductor [24]. Twelve probes in liver and four probes in adipose tissue were differentially expressed between GK and BN rats at a nominal p < 0.05 significance level (Table 2). Following FDR correction for multiple testing (for 170 probes) none of these remained significant, likely due to the small sample size (4 arrays per tissue). MiR-125a was the most significant result for GK vs. BN in liver (FC = 5.62, *P *= 0.001, *P*_*adjusted *_= 0.10)), and we confirmed this over-expression of miR-125a by RT-PCR (FC = 13.15, two-sided t-test *P *= 0.0005) (Figure 1). The top-ranking miRNA in adipose tissue was miR-322 (FC = 1.91, *P *= 0.029 *P*_adjusted _= 0.99) but it did not rank highly in the liver. MiR-29a, which ranked fourth in the adipose GK vs. BN comparison, has been previously reported to be over-expressed in adipose tissue in GK rats when compared to normoglycaemic Wistar controls [19]. The only miRNA that showed some evidence of change in expression in both liver and adipose tissue is miR-125a, which ranked top in the liver and fifth in the GK vs. BN comparison in adipose tissue (FC = 1.97, *P *= 0.078, *P*_*adjusted *_= 0.99).

**Table 2:** MiRNA microarray analysis reveals differential expression between hyperglycaemic and normoglycaemic rats

**Rank**	**miRNA**	**Fold change** **(GK vs. BN)**	**P-value**	**Adjusted** **P-value**
**Liver**				
1	miR-125a	5.61	0.001	0.104
2	miR-30e	1.56	0.009	0.522
3	miR-210	1.56	0.013	0.522
4	miR-221	1.60	0.015	0.522
5	miR-223	1.50	0.015	0.522
6	miR-365	1.41	0.029	0.592
7	miR-26b	1.51	0.031	0.592
8	miR-23a	2.30	0.032	0.592
9	miR-222	1.38	0.033	0.592
10	miR-29c	1.52	0.038	0.592
11	miR-30a-3p	1.32	0.045	0.592
12	miR-22	-1.39	0.050	0.592

**Adipose tissue**				
1	miR-322	1.91	0.029	0.999
2	let-7c	-2.27	0.040	0.999
3	let-7b	-2.17	0.046	0.999
4	miR-29a	1.51	0.049	0.999
5*	miR-125a	1.97	0.078	0.999

Differences in miRNA expression levels in 4 hyperglycaemic GK rats compared to 4 normoglycaemic BN rats in liver and adipose tissue. For each tissue, one GK and one BN sample were hybridized to each array in a two-colour experiment (4 arrays per tissue). M-ratios therefore represent the log_2 _intensity ratio in the two strains. The limma package from BioConductor was used to find miRNAs differentially expressed between GK and BN rats and the top ranking genes for each tissue are shown. * miR-125a is shown in this table to illustrate the potential overlap of signals detected in liver and adipose tissue.

### Differences in gene (mRNA) expression between GK and BN rats

**Figure 2:**
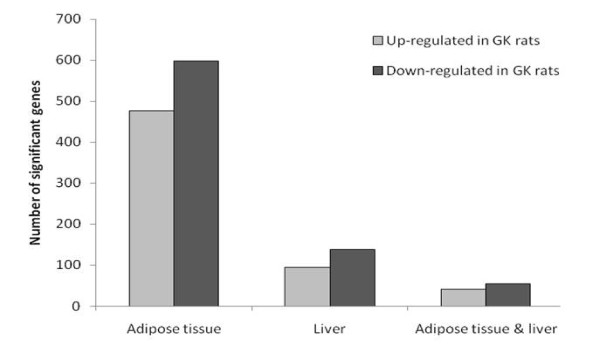
**Gene expression analysis of adipose tissue and liver reveals differential expression between hyperglycaemic and normoglycaemic rats**. The number of genes that are significant (adjusted p < 0.05) in the comparison of 4 hyperglycaemic GK rats and 4 normoglycaemic BN rats in adipose tissue (477 up; 598 down), liver (95 up; 138 down), and in both tissues (41 up; 55 down). Five genes significant in both tissues showed opposite directions of change and are not included in the adipose tissue and liver group.

Gene expression levels in adipose tissue and liver from the same rats used in the miRNA experiment were analysed using the Illumina Rat Ref12 array. We identified 1075 genes differentially expressed (adjusted p < 0.05) in adipose tissue in the hyperglycaemic GK rat compared to the normoglycaemic BN rat (477 up-regulated and 598 down-regulated). In liver, we found 233 differentially expressed genes, of which 95 were up regulated in GK rats and 138 were down regulated (Figure 2). A total of 101 genes were significantly altered in both adipose tissue and liver, with the vast majority showing the same direction of change in both tissues (41 up and 55 down). The robustness of gene differential expression is supported by the previously replicated array experiments using two well established and technologically different platforms using RNA from the current samples [37]. *In-silico *functional profiling of the significant genes revealed similar results in both tissues (Table 3), including several pathways related to glucose lipid metabolism: prostaglandin and leukotriene metabolism (*P*_*adjusted *_= 0.001 in adipose tissue, *P*_*adjusted *_= 0.002 in liver), glycolysis/gluconeogenesis in adipose tissue (*P*_*adjusted *_= 0.003) and fatty acid metabolism in liver (*P*_*adjusted *_= 0.01). A number of amino acid metabolism categories were also significant in both tissues (Table 3). (Details of individual genes for all significant categories are given in [Additional file 5]).

**Table 3:** Functional profiling of differentially expressed genes in GK compared to BN rats in adipose tissue (n = 1075) and liver (n = 233) using GENECODIS.

**KEGG pathway(s)**	**Number of genes**	**Corrected p-value**
***Adipose tissue***		
Prostaglandin and leukotriene metabolism	10	0.001
Focal adhesion | ECM-receptor interaction | Hematopoietic cell lineage | Regulation of actin cytoskeleton	4	0.002
ECM-receptor interaction | Hematopoietic cell lineage	5	0.002
ECM-receptor interaction	11	0.002
Glycolysis/Gluconeogenesis	9	0.003
Focal adhesion | ECM-receptor interaction	9	0.003
Arginine and proline metabolism	8	0.005
Glycolysis/Gluconeogenesis | Fructose and mannose metabolism	4	0.005
Glycolysis/Gluconeogenesis | Pentose phosphate pathway | Fructose and mannose metabolism	3	0.012
Hematopoietic cell lineage	9	0.015
Cell Communication | Focal adhesion | ECM-receptor interaction	6	0.019
Fructose and mannose metabolism	6	0.019
Porphyrin and chlorophyll metabolism	5	0.029
Folate biosynthesis	5	0.031
Metabolism of xenobiotics by cytochrome P450	7	0.033
Cytokine-cytokine receptor interaction | Focal adhesion	3	0.033
Focal adhesion	14	0.033
Valine, leucine and isoleucine degradation	6	0.033
Cholera – Infection	5	0.033
Fructose and mannose metabolism | Galactose metabolism	3	0.033
Urea cycle and metabolism of amino groups	4	0.033
Glycine, serine and threonine metabolism	5	0.044

***Liver***		
Arginine and proline metabolism	7	2.17E-06
Alanine and aspartate metabolism | Arginine and proline metabolism	3	2.09E-05
Alanine and aspartate metabolism	4	1.30E-04
Urea cycle and metabolism of amino groups | Arginine and proline metabolism	3	0.0017
Prostaglandin and leukotriene metabolism	4	0.0019
Valine, leucine and isoleucine degradation | Butanoate metabolism	3	0.0030
Fatty acid metabolism	3	0.0103
ECM-receptor interaction	3	0.0361

### Predicted targets for miR-125a and *in-silico *functional profiling

The miRNA target gene prediction algorithms TargetScan 4.1 [38], PicTar [31] and miRanda [32] generated target gene lists for miR-125a comprising 165, 350 and 975 genes, respectively. Of these, 152 genes were predicted by more than one algorithm [Additional file 3] and only 17 are predicted by all algorithms. The target gene lists were analysed using the GENECODIS tool to investigate the biological pathways (KEGG) that may be affected by overexpression of miR-125a in liver and, to a lesser extent, adipose tissue. When the set of 152 miR-125a target genes was tested, mitogen-activated protein kinase (MAPK) signaling was the only pathway to show significant over-representation (*P*_*adjusted *_= 3 × 10^-5^). The MAPK signaling pathway was also significant when each predicted target list was analysed separately, and was the top-ranking pathway for the lists generated by TargetScan and PicTar [Additional file 6]. Other significant pathways of note included insulin signaling (*P*_*adjusted *_= 0.03, PicTar gene list) and, with the miRanda list, glycerolipid metabolism (*P*_*adjusted *_= 0.02) and calcium signaling (*P*_*adjusted *_= 0.05).

### Enrichment of miR-125a target genes among differentially expressed genes

Given the evidence for miRNA regulation at the mRNA level in animals [6,9,10], integration of miRNA-mRNA expression data was also performed. Since miR-125a was the top-ranking miRNA in liver, and fifth-ranked in adipose tissue, we hypothesized that target genes of miR-125a would be differentially expressed between GK and BN rats in both tissues. Specifically, overexpression of miR-125a in these tissues in the GK rat would be expected to down-regulate miR-125a target genes [1,6,39]. Target genes predicted by all algorithms (n = 17), plus the set of 152 genes predicted by more than one algorithm, were tested for enrichment among the 598 genes significantly down-regulated in GK compared to BN rats in adipose tissue and among the 138 down-regulated genes in liver (Table 1). Greater overlap than expected by chance (see Methods) was observed in adipose tissue (overlap = 22, FDR = 0.04) but not in liver (overlap = 5, FDR = 0.25) for the set of 536 target genes predicted by the miRanda algorithm and present on the Illumina RatRef 12 array. Three miR-125a target genes (*Slc35c2*, *Umps *and *Ptges2*) were significantly down-regulated in GK rats in both tissues. Contrary to expectation, a significant enrichment was also observed when the up-regulated genes were tested (overlap = 21, FDR = 0.006 in adipose tissue; overlap = 5, FDR = 0.07 in liver). For both up- and down-regulated genes, there was more overlap in adipose tissue compared to liver, even though miR-125a showed a higher fold induction in liver.

Neither of the target gene lists generated by PicTar and TargetScan, nor the combined list, showed significant overlap with differentially expressed genes (Table 1). This was because the overlap between differentially expressed genes and miR-125a targets occurred largely for genes uniquely predicted by miRanda. Furthermore, the PicTar and TargetScan lists are more similar to each other than to the miRanda list, and so the combined list is biased toward the genes predicted by these algorithms [Additional file 3]. It should be noted that it is not the greater number of targets predicted by miRanda that produces the significant result, as the size of the target gene list is accounted for in the analysis.

## Discussion

The role of altered miRNA expression in a variety of diseases is increasingly being recognized but the precise nature and downstream effects of such changes is largely unknown. Previous studies have shown that miRNAs regulate various physiological events relevant to T2D pathophysiology, such as insulin secretion, insulin responsiveness and energy homeostasis. Here, we have characterized differential miRNA and mRNA expression in insulin sensitive tissues of rats of a model of T2D (GK rat) and in normoglycaemic BN controls, a strain combination extensively used to study the genetic determinants of T2D phenotypes in F2 hybrids and congenic strains [21]. In the miRNA data, the most striking finding was the over-expression of miR-125a in both liver and adipose tissue (with nearly 6-fold and 2-fold higher expression, respectively) in GK compared to BN rats. This finding was validated by RT-PCR in liver (FC = 13.15, p = 0.0005). The higher sensitivity and dynamic range of the RT-PCR technique is the most likely reason why the fold change is higher than detected by microarray.

MiR-125a and its close homolog miR-125b differ by a single nucleotide [40], and thus share many predicted target genes. Experimentally validated target genes of both miR-125a and miR-125b in humans include ERBB2 and ERBB3 [41] and LIN28 [42]. Both miR-125a and miR-125b have been reported to be down-regulated in ovarian [43] and breast cancers [44], with potential roles in cell proliferation and differentiation. To further investigate the potential role of miR-125a in T2D, we assessed the functional roles of predicted miR-125a target genes. This analysis suggested that increased miR-125a levels may particularly affect genes involved in the MAPK signalling pathway. MAPK has been implicated in T2D [28,30] and plays a critical role in insulin signalling [45], and may contribute to insulin resistance [46]. Further functional experiments will help elucidate the role of MAPK signalling in T2D and to what extent increased miR-125a expression may affect this pathway.

We integrated our miRNA results with gene expression data from the same animals and found an enrichment of miR-125a target genes predicted by the miRanda algorithm [32] among genes down-regulated in GK rats in adipose tissue. Of particular interest, a proposed functional candidate gene for T2D, Ptges2, is a predicted miR-125a target gene and was significantly down-regulated in both tissues. Two other miR-125a target genes of interest in the context of T2D and obesity are *Ppap2c *and *Sult1a1*, both of which were significantly down-regulated in adipose tissue from the GK rat. In addition; single nucleotide polymorphisms (SNPs) in the *SULT1A1 *region have previously been associated with increased BMI in humans [47]. Contrary to expectation, it was surprising to find an even stronger enrichment among the up-regulated genes. One possibility is that these are not direct target genes themselves, but represent changes further downstream of the miRNA regulation. No enrichment was observed with the target gene lists predicted by the other two algorithms, which may be due to differences in the target-gene prediction methods implemented or to the effects of long-term exposure to hyperglycaemia further affecting the control of gene expression. These results show that the relationship between miRNAs and gene expression is not a simple one.

Though the enrichment of miR-125a target genes predicted by miRanda is an interesting finding, it raises a number of questions, including why both down and up-regulated genes should show significant enrichment of miR-125a target genes, and why stronger enrichment was observed for the tissue with lower fold induction of miR-125a. The difficulty of predicting genuine target genes combined with other influences on gene expression could provide some explanation. Furthermore, the current data do not demonstrate whether the expression of any of the genes is dysregulated as a direct consequence of the increased miR-125a levels. One approach to identify genes likely to be regulated by miR-125a is to find those that show a strong negative correlation between their expression and miR-125a levels. Unfortunately, the two-colour experimental design used for the miRNA study (generating GK v BN expression ratios) combined with the small sample size precluded such an analysis here. Integrating miRNA and mRNA data has the potential to give further insights into miRNA-mediated regulation of gene expression but is limited to detecting events in which the target mRNA is degraded. Still, in-silico approaches will likely be useful for prioritizing target genes for functional validation.

Our results suggest that increased miR-125a expression may be a characteristic feature of hyperglycaemic GK rats. However, as this study was carried out in rats with long-established T2D phenotypes, it remains unclear whether the observed changes are causative, or reflect adaptation to prolonged hyperglycaemia. GK and BN rats are also genetically different strains, and it is therefore possible that strain differences unrelated to hyperglycaemia could contribute to the altered expression of miR-125a. Although *in-silico *functional profiling of gene expression data and miR-125a target genes lends some support to the idea that miR-125a over-expression is linked to the hyperglycaemic phenotype, further evidence from other model systems or functional studies is certainly desirable.

Among the other miRNAs showing robust differences between GK and BN rats was a 1.5 fold up-regulation of miR-29a in adipose tissue. This is exactly comparable with the only previous study of miRNAs in T2D, which identified a 1.5 fold up-regulation of miR-29a, together with the paralogs 29b and 29c, in skeletal muscle from GK and Wistar rats (normoglycaemic controls)[19]. Northern blot analysis confirmed the up-regulation of all three miR-29 paralogs in muscle, adipose tissue and liver. This study further showed that high glucose and insulin (which indicate insulin resistance) up-regulate the expression of miR-29a/b in adipocyte cell lines. They also suggest the over-expression of miR-29a/b inhibits insulin-induced glucose import in the same adipocyte cell lines. Taken together, these data support the involvement of miR-29a in insulin resistance and the insulin-signaling pathway.

## Conclusion

We have shown that miR-125a expression is increased in two insulin target tissues in a rat model of T2D. Gene expression analysis in the same animals revealed a distinct profile characterizing the hyperglycaemic GK rat, including altered expression of several predicted miR-125 target genes. *In-silico *functional profiling of predicted miR-125a targets and differentially expressed genes indicated that lipid metabolism pathways and MAPK signaling may be dysregulated in the hyperglycaemic state. Knowledge of miRNA differential expression between GK and BN rats provides important information which may contribute to the understanding of processes which underlie the T2D phenotype in GK rats through investigations in crosses and congenic models derived in this strain combination [48-50]. Further studies at the protein level and translation across species, especially to humans, will be important to elucidate the potential role of miR-125a in T2D pathophysiology.

## Competing interests

The authors declare that they have no competing interests.

## Authors' contributions

BMH and HL performed statistical analysis, validated miRNA expression and drafted the manuscript. CML, JMT and QFW contributed with statistical analysis, and data interpretation. PK, AB and CF participated in growing animal colonies, tissue and RNA extraction and mRNA sample preparation for microarrays. CC and JR generated and scanned Ambion Microarrays. DG, MIM and CML designed the study and coordinated it and finalized the manuscript. All authors read and approved the final manuscript.

## Pre-publication history

The pre-publication history for this paper can be accessed here:



## Supplementary Material

Additional file 1 **Outline of methodology for analysis and integration of miRNA and mRNA expression data from hyperglycaemic GK and normoglycaemic BN rats.** Click here for file

Additional file 2 Results of intra-peritoneal glucose tolerance test (IPGTT) measurements carried out at four-months of age on animals representative of the colony GK = 8 BN = 4 *P < 0.05, **P < 0.01, ***P < 0.001 significance. Click here for file

Additional file 3 **Overlap of target-gene lists predicted for miRNA rno-miR-125a using three algorithms.** Click here for file

Additional file 4 **Tissue differences in miRNA expression (liver vs. fat)** Click here for file

Additional file 5 **Functional profiling and gene-symbols of differentially expressed genes in GK compared to BN rats in adipose tissue (n = 1075) and liver (n = 233) using GENECODIS** Click here for file

Additional file 6 **In-Silico functional profiling of miR-125a targets predicted by miRanda (worksheet 1), TargetScan (worksheet 2), and PicTar (worksheet 3).** Click here for file
