# Supplementary figures and images for: MicroRNA-125a is over-expressed in insulin target tissues in a spontaneous rat model of Type 2 Diabetes
Source: BMC Med Genomics. 2009 Aug 18;2:54. doi: 10.1186/1755-8794-2-54 (PMC2754496; doi:10.1186/1755-8794-2-54)

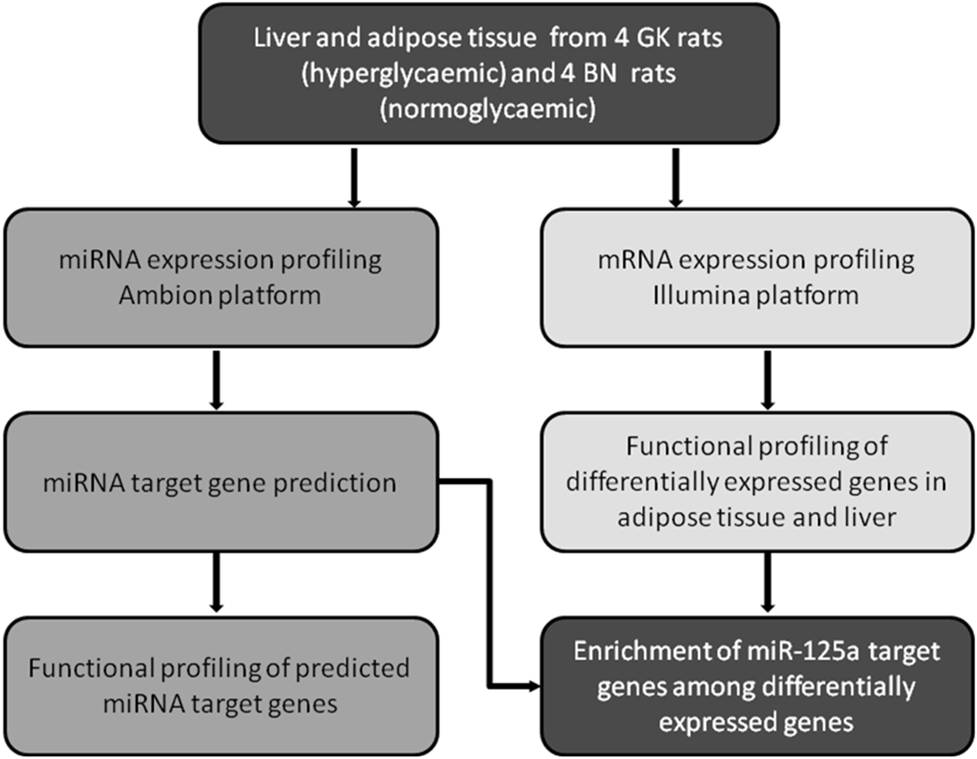

Supplement: Additional file 1 — Outline of methodology for analysis and integration of miRNA and mRNA expression data from hyperglycaemic GK and normoglycaemic BN rats. [file 1755-8794-2-54-S1.jpeg]

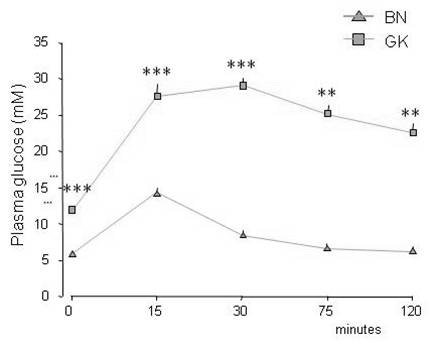

Supplement: Additional file 2 — Results of intra-peritoneal glucose tolerance test (IPGTT) measurements carried out at four-months of age on animals representative of the colony GK = 8 BN = 4 *P < 0.05, **P < 0.01, ***P < 0.001 significance. [file 1755-8794-2-54-S2.jpeg]

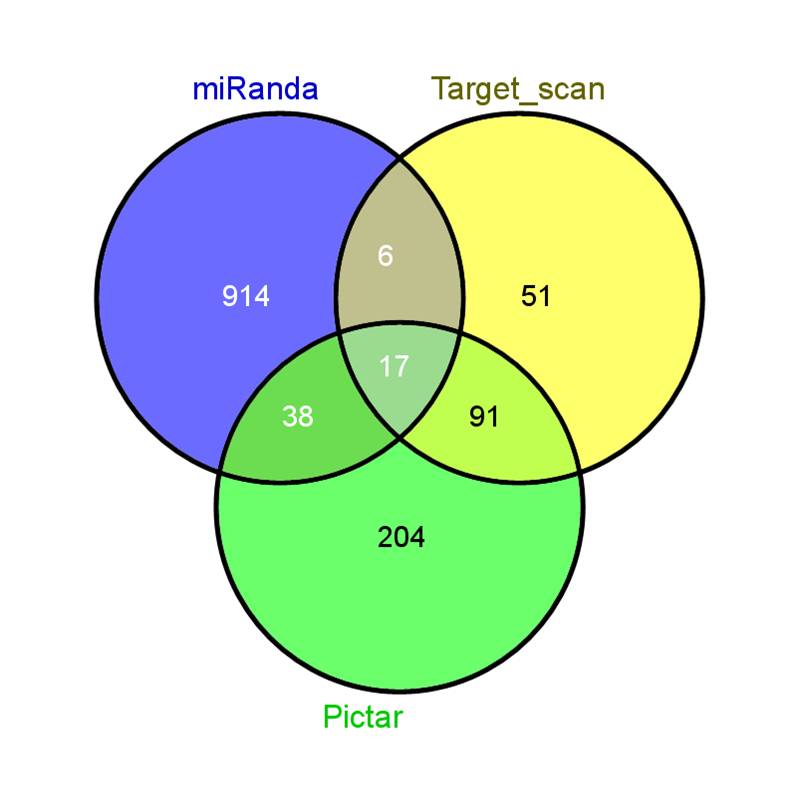

Supplement: Additional file 3 — Overlap of target-gene lists predicted for miRNA rno-miR-125a using three algorithms. [file 1755-8794-2-54-S3.jpeg]
